# Anti-IgE: A treatment option in allergic rhinitis?

**DOI:** 10.5414/ALX02205E

**Published:** 2021-02-24

**Authors:** Oliver Pfaar, Francesca Gehrt, Hansen Li, Stefan A. Rudhart, Alexander Nastev, Boris A. Stuck, Stephan Hoch

**Affiliations:** * The two first authors have equally contributed to the publication; Department of Otorhinolaryngology, Head and Neck Surgery, Section of Rhinology and Allergy, University Hospital Marburg, Philipps-Universität Marburg, Marburg, Germany

**Keywords:** omalizumab, biologics, allergic rhinitis, anti-IgE, allergen immunotherapy

## Abstract

Background: Allergic rhinitis (AR) is the most common IgE-mediated allergic disease. Multiple clinical trials have demonstrated promising results on the AR treatment with biologics, in particular with the use of omalizumab – an anti-IgE antibody. Omalizumab has also been established in the routine management of allergic asthma and chronic idiopathic urticaria. However, currently there is no approved license for the use of biologics in AR in Germany. Materials and methods: A systematic literature review has been completed including randomized controlled trials, meta-analyses, and reviews on the treatment of AR with omalizumab. Results: The systematic review demonstrates strong evidence supporting the use of omalizumab in the treatment of AR with regard to symptom control, safety profile, and management of comorbidities. Conclusion: Omalizumab is a good and safe option in the treatment of AR in terms of symptom control and the management of pre-existing comorbidities. Further clinical trials with other biologics in the management of AR are needed and are expected to follow soon.

## Introduction 

[Table Abbreviation]Allergic rhinitis (AR) is an IgE-mediated chronic disease of the nasal mucosa. As a result of exposure to allergens, typical symptoms such as rhinorrhea, nasal obstruction, sneezing, or itching in the area of the nose occur [1]. 

In Germany, AR is the most common allergic disease with a lifetime prevalence of 14.8%, as epidemiological studies of the Robert Koch Institute (RKI) have demonstrated [2]. In addition, an overall increase of the prevalence of AR has been reported worldwide [3]. Nevertheless, it is often misdiagnosed, under-diagnosed, and underestimated as such [4]. The standard treatment of AR is based on three principles: i) avoidance measures, ii) anti-allergic pharmacotherapy, and iii) allergen immunotherapy (AIT) [1, 5, 6]. Nevertheless, in many patients, complete symptom control cannot be achieved with the established standard therapies, and quality of life and (work) productivity are often severely affected and impaired. 

In particular, sufficient efficacy of avoidance measures cannot always be realized. Furthermore, the administration of anti-allergic medication cannot provide a thorough symptom relief in all patients. This is especially true for nasal obstruction under antihistamine therapy [7, 8]. There is moreover an association of AR and relevant comorbidities such as allergic asthma and rhinosinusitis, which additionally impairs patients’ conditions [4, 9, 10, 11]. 

## Biologics and modulation of the inflammatory cascade 

The former underlines the important unmet need for further improvement of therapeutic approaches. Among others, biologicals are promising therapeutic candidates directly targeting individual mechanisms of the inflammatory cascade. Between 1997 and 2019, a large number of phase I and II studies were conducted on efficacy and safety of biologics in AR (Table 1). A first group of biologics comprise monoclonal antibodies (MAB) against IgE, such as talizumab, MEDI4212, ligelizumab, XmAb7195, and omalizumab. Though talizumab (CGP51901) has demonstrated a blunting of serum IgE levels in preliminary studies, further clinical development programs have been paused [12]. In an early phase I trials of MEDI4212, a particularly rapid and strong reduction in serum IgE was observed in patients with AR followed by a rapid rebound of serum IgE levels after cessation of therapy [13]. Ligelizumab also demonstrated a significant reduction of serum IgE in a phase I study which could be maintained for a longer period after treatment [14]. 

A second class of biologics targets interleukins (IL) such as IL-4 and IL-13, and has been investigated in AR patients. In a phase II study, a combined therapy with VAK-694 (IL-4 antagonist) and subcutaneous allergen immunotherapy (SCIT) demonstrated an immunomodulatory effect on the TH2 memory cells; however, a clinical response was lacking [15]. In contrast, dupilumab targeting both the IL-4 and IL-13 receptors showed a significant improvement in symptoms in patients with persistent AR (PAR) and comorbid asthma [16]. Furthermore, the toll-like receptor 7 (TLR-7) agonist GSK2245035 was found to be beneficial in AR in a phase II study. However, further dose-ranging trials are needed for detecting the optimal dosage [17]. 

Currently, the most advanced biologic in AR in clinical development is the IgE antagonist omalizumab. It has been approved in the EU since 2005 as an add-on therapy for patients with severe persistent allergic asthma and has been established in routine clinical care [19]. However, due to its mechanism of action, omalizumab is also considered a promising therapeutic option for the treatment of other IgE-mediated atopic diseases such as AR [20]. This is probably also true for its biosimilars currently in development (CMAB007, STI-004, FB317, GBR310, CTP-39, BP001) [12]. The following is an overview of the current literature on efficacy and safety of omalizumab in AR. 

## Omalizumab in AR: search strategies 

**Figure 1.**
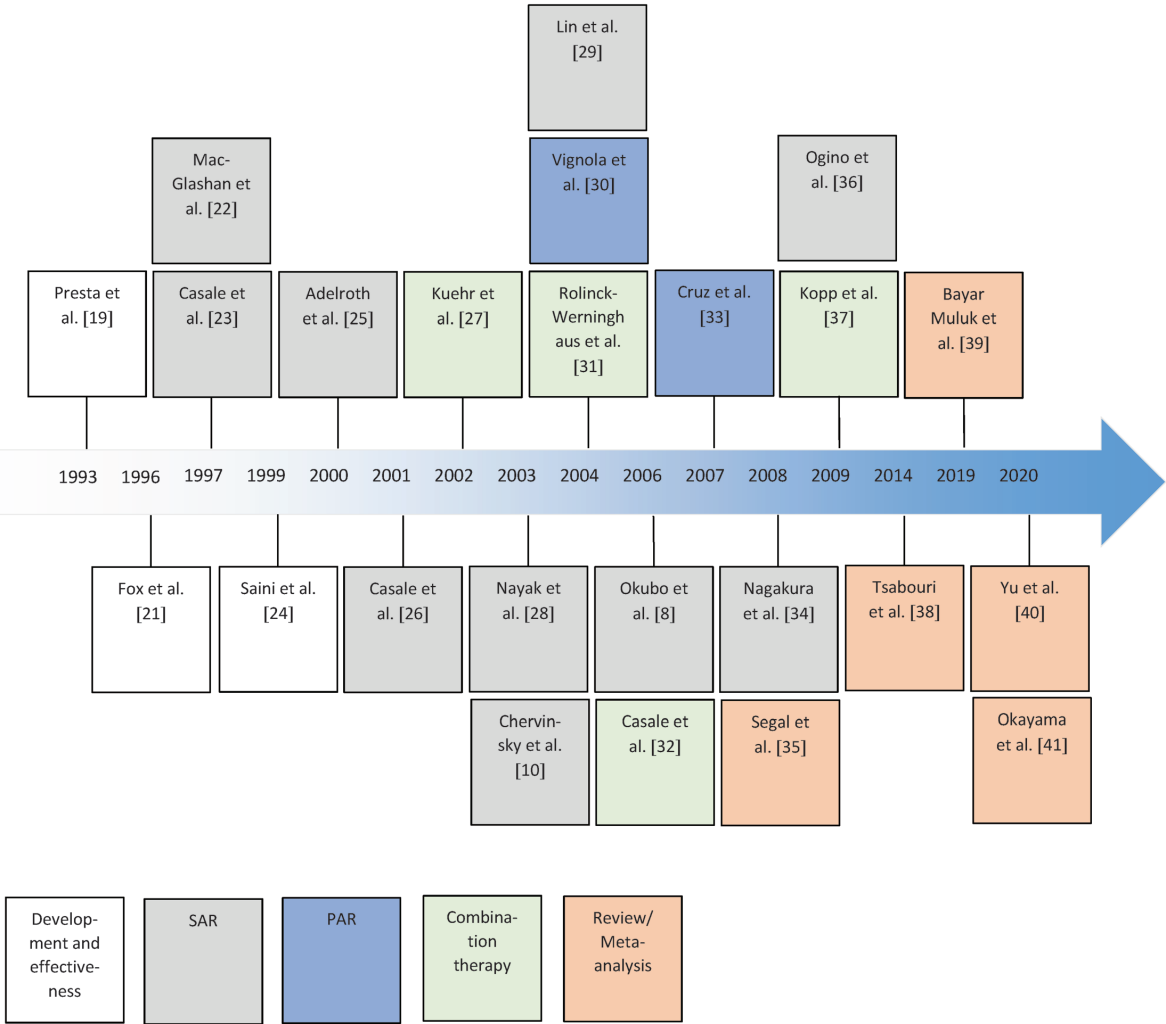
Clinical trials of omalizumab in AR. SAR = seasonal allergic rhinitis; PAR = perennial allergic rhinitis.

The literature search was conducted using PubMed, clinicaltrialisregister.eu, and clinicialtrialis.gov. The search terms “biologicals”, “omalizumab”, and “anti-IgE” in combination with “allergic rhinitis” were used to identify relevant study results. Randomized controlled trials (RCT), placebo controlled (DBPC) trials, review articles, and meta-analyses from 1997 to 2020 were considered for the literature search. The most recent literature search was conducted on April 27, 2020 and aimed to specify the effect of omalizumab on symptom control, reduction of concomitant (anti-allergic) medications for symptom relief, disease-specific quality of life, safety of SCIT under omalizumab and safety/tolerability in general. Figure 1 provides an overview of trials on omalizumab in AR extracted by our search. 

## Omalizumab in AR: mechanism-profile 

As in other atopic diseases, allergen-specific IgE is overexpressed in patients with AR binding to two different receptors (FcεRI and FcεRII) on effector cells. The binding to these receptors results in the initiation of the inflammatory cascade with the typical pattern of AR symptoms. Increased levels of serum allergen specific IgE have been found to correlate with the onset and symptom severity of AR [26, 42]. 

Omalizumab binds free serum IgE, preventing the interaction of IgE with the FcεRI receptor and further activation of the subsequent inflammatory pathways. Omalizumab has a higher affinity to free IgE in the serum than the FcεRI receptor of effector cells leading to a strong reduction in free circulating IgE [43]. This is followed by a migration of mast cell and basophilic granulocytes recruitment and activation [39] suppressing the inflammatory pathways within the nasal mucosa after allergen exposure [44]. 

However, it should be noted that omalizumab, when given at therapeutic doses, does not bind IgE that is already bound to the FcεRI or FcεRII receptor [41]. Since basophilic granulocytes and mast cells have a high binding capacity for IgE, a strong reduction of free IgE and FcεRI receptors is necessary to reduce the histaminergic response of effector cells [45]. In clinical trials on omalizumab, serum free IgE concentrations were reduced by 84 – 99%, degranulation by up to 90%, and FcεRI receptor expression on basophilic granulocytes by 73 – 99% [2^1^, ^22^, 24, 29, 30, 46]. 

Previous study results also indicate that omalizumab not only suppresses the acute inflammatory response in allergic rhinitis, but also may provide a long-term immunomodulatory effect [26]. 

## Omalizumab in AR: practical aspects 

### Dosage 

Early studies on the use of omalizumab in AR aimed to determine the optimal dosage. Casale et al. [47] compared doses between 50 and 300 mg every 3 – 4 weeks over a period of 8 – 9 weeks with placebo administration. The 300-mg group was significantly superior compared to the placebo group. However, symptom improvement was also seen in patients receiving lower doses. The manufacturer (Novartis Pharmaceuticals, East Hanover, NJ, USA) recommends a dose of 0.016 mg/kg/IgE (IU/mL) subcutaneously every 4 weeks in adults and adolescents (12 years of age and older) for the treatment of severe persistent asthma [35]. Recent trials in AR with this dosage could demonstrate clinical efficacy [8, 10, 27, 3^2^, ^34^, ^37^]. 

### Omalizumab: patient profiles in AR 

Omalizumab has been demonstrated to be beneficial for patients with moderate to severe intermittent and persistent AR and insufficient symptom control despite conventional therapy [38]. Several large clinical studies reported a significant improvement in symptom control, reduction in the use of concomitant anti-allergic medication, and an improvement in the quality of life [10, 23, 26, 38, 39]. Of note, the baseline values of patients with AR seem to correlate with the treatment effect size of omalizumab, indicating moderate effects in lower IgE profiles [48]. However, omalizumab is not approved in Germany in this indication. 

Further atopic diseases such as allergic asthma and urticaria are often associated with AR [10], which often aggravates these conditions [26]. 

According to a variety of studies, about half of all patients with AR also suffer from allergic asthma, and concomitant AR has a significant impact on asthma control. Poorly controlled AR is associated with a higher number of asthma exacerbations and hospitalizations [49]. Since both atopic diseases are associated with elevated serum IgE levels, efficacy of IgE-lowering measures is obvious. In a study by Vignola et al. [30] on the efficacy and safety of omalizumab in patients with comorbid asthma and AR, a significant improvement in asthma and rhinitis symptoms as well as lung function was observed. Furthermore, an improvement on patients’ quality of life was demonstrated [30]. 

In addition to trials investigating efficacy of omalizumab monotherapy, some studies combined this treatment with AIT aimed to increase treatment response to AR patients [31, 37, 38, 39]. In a clinical study, Kuehr et al. [27] were able to demonstrate a 48% better symptom control with combination therapy compared to AIT as a monotherapy, indicating a clinical benefit of this add-on co-treatment. 

### Reduction of rhinoconjunctival symptoms 

Various endpoints have been used in clinical studies to assess symptom severity. The most common score used to measure nasal symptoms was the Daily Nasal Symptom Severity Score (DNSSS). A large number of studies also considered the ocular symptoms of allergic rhinoconjunctivitis (ARC) such as the Daily Ocular Symptom Severity Score (OCSSS) [8, 10, 25, 34, 50]. 

A meta-analysis from 2014 by Tsabouri et al. [38] evaluated nine randomized controlled trials with regards to the effect of omalizumab on the DNSSS in AR and could demonstrate superiority against placebo during the pollen season in SAR [25, 50]. A study by Chervinsky et al. [10] on PAR also showed significantly more symptom improvement in the omalizumab group compared to the placebo group. In this study, 28% of patients treated with omalizumab achieved symptom control, compared with only 10% of patients in the control group [24]. Another study by Nagakura et al. [34] also demonstrated significant superiority of omalizumab over an anti-allergic drug (Th2 cytokine inhibitor). 

In general, a significant improvement in nasal symptoms was observed in all randomized controlled trials between 2000 and 2010. A particularly strong treatment effect size of 39% was seen when omalizumab was combined with AIT [30, 37]. A meta-analysis published in 2020 [40] again confirmed the benefit of omalizumab by evaluating 9 original studies regarding nasal symptoms and 5 studies regarding ocular symptoms. A significant reduction in symptoms was demonstrated for both SAR [25, 50] and PAR [10]. 

However, the extent of symptom reduction varied between the studies. For example, Adelroth et al. [25] observed a smaller effect of omalizumab on OCSSS than on DNSSS, whereas the reduction in ocular symptoms (50%) exceeded the improvement of nasal symptoms (40%) in the study by Okubo et al. [8]. 

### Reducing the need for anti-allergic medication 

While the study designs were consistently uniform in recording of nasal and ocular symptoms, there were notable methodological differences in the use of concomitant anti-allergic medications. For example, in some studies, this medication was paused following the study protocol [10], whereas allowed in others [34]. Depending on the study, different anti-allergic medication was provided for treatment of AR symptoms, such as systemic and topical antihistamines, mast cell stabilizers, naphazoline, and topical and systemic corticosteroids [8, 10, 25]. However, an anti-allergic medication sparing effect of ~ 50% was observed with omalizumab therapy in several randomized controlled trials [25]. Furthermore, Chervinsky et al. [10] and Okubo et al. [8] reported a reduction of days on which an anti-allergic medication had to be taken by a third and to a fifth, respectively, while no significant differences were observed in the placebo group. It should be noted, however, that other studies came to a different conclusion [27, 30]. 

### Impact of omalizumab on quality of life 

To assess the impact of omalizumab therapy on quality of life, the standardized and validated Rhinoconjunctivitis Quality of Life Questionnaire (RQLQ) has frequently been used in AR trials. This questionnaire contains 28 items relating to activity limitations, sleep disturbances, symptoms, practical problems, and emotional functioning [10]. Adelroth et al. [25] and Chervinsky et al. [10] found a statistically significant improvement in the omalizumab group compared to the placebo group in all seven domains of the RQLQ and in the total score using this questionnaire. Even in patients who did not previously respond to AIT or intranasal steroid treatment, a significant improvement in the total score was demonstrated [10]. These findings are further supported by a recent meta-analysis by Yu et al. and others [10, 25, 26, 39, 40, 51]. 

### Safety aspects of omalizumab 

Omalizumab therapy has been shown to be safe and well tolerated in routine clinical care. This is also confirmed in a meta-analysis by Yu et al. [40] which found no significant difference in the frequency of adverse events between the omalizumab and placebo groups as also reported by other authors [12, 49]. Due to the lack of interaction of omalizumab with cell-bound IgE, the risk of anaphylaxis is generally considered to be low [34]. This is supported by the results of the Omalizumab Joint Task Force (OJTF), an initiative of the American Academy of Allergy, Asthma & Immunology. The data analysis of the OJTF showed that the probability of an anaphylactic event after omalizumab administration is ~ 0.09% [52]. The majority of anaphylactic reactions occur in the first 120 minutes and within the first 3 injections. Therefore, patients should be followed 30 minutes after each injection, especially as possible causes and predisposing factors for anaphylactic and anaphylactoid reactions are still unclear. A case-control study in 2016 identified previous anaphylaxis and food allergy as possible risk factors [53]. In general, anaphylactic reactions were rare in clinical trials [5, 29]. Studies on long-term treatment with omalizumab in PAR also showed good tolerability [1]. There seems to be no increased risk of adverse events including anaphylactic reactions in repetitive treatment cycles [28, 36]. 

In rare cases, antibodies to the drug may be formed during therapy with humanized DNA-derived monoclonal antibodies in general. However, such events have not been observed with omalizumab [1, 29, 34]. 

Omalizumab is able to form an immune complex with free IgE. Since these complexes are present as trimers or hexamers, they pose a low risk for the development of serum sickness due to their small size [54]. Based on available data, a low risk for the occurrence of serum sickness can be assumed [54, 55]. So far, only a few cases have been described in the literature. 

Large clinical studies showed no increased risk for the occurrence of malignant tumors with omalizumab [56]. In animal studies, omalizumab in higher doses sometimes caused pronounced changes in the blood count, such as thrombocytopenia. In the more recent clinical studies, neither the occurrence of such blood count changes nor the formation of antibodies against omalizumab could be observed [8, 10]. An evaluation of twelve randomized controlled trials could also not determine any clinically relevant blood count changes under omalizumab treatment [55]. 

A clinical study demonstrated a slight increase in parasitic infections under anti-IgE therapy [33]. However, an increase in parasitic infections could not be reproduced in the animal model. In fact, low IgE blood serum concentrations showed no influence on the course of parasitic infections or the risk of reinfection [57, 58]. Clinical trials of asthma therapy with omalizumab also did not show an increased incidence of parasitic infections, nor did they show refractory courses of anti-parasitic treatment. [54]. 

## Conclusion 

In the treatment of AR, monoclonal antibodies to IgE represent a promising therapeutic option for the future by effectively reducing the free IgE antibodies in the serum. 

Omalizumab has already been the subject of a large number of clinical studies in AR with supportive evidence from the treatment of other atopic diseases. In AR, this treatment has demonstrated clinical efficacy by reducing the symptomatic burden and need for concomitant allergic medication and by improving the quality of life in patients with AR. In addition, it has an impact on comorbidities and a convincing safety profile. 

Omalizumab is approved as an add-on therapy for better control of severe persistent allergic asthma, the severe form of chronic rhinosinusitis with polyposis (CRSwNP) and the severe form of chronic spontaneous urticaria. Clinical development programs in AR are also very convincing. However, further clinical studies on omalizumab and other above-mentioned biologics are needed to integrate these molecules in common treatment concepts of AR. 

## Funding 

None. 

## Conflict of interest 

Oliver Pfaar reports grants and personal fees from ALK-Abelló, grants and personal fees from Allergopharma, grants and personal fees from Stallergenes Greer, grants and personal fees from HAL Allergy Holding B.V./HAL Allergie GmbH, grants and personal fees from Bencard Allergie GmbH/Allergy Therapeutics, grants and personal fees from Lofarma, grants from Biomay, grants from Circassia, grants and personal fees from ASIT Biotech Tools S.A., grants and personal fees from Laboratorios LETI/LETI Pharma, personal fees from MEDA Pharma/MYLAN, grants and personal fees from Anergis S.A., personal fees from Mobile Chamber Experts (a GA2LEN Partner), personal fees from Indoor Biotechnologies, grants and personal fees from Glaxo Smith Kline, personal fees from Astellas Pharma Global, personal fees from EUFOREA, personal fees from ROXALL Medizin, personal fees from Novartis, personal fees from Sanofi-Aventis and Sanofi-Genzyme, personal fees from Med Update Europe GmbH, personal fees from streamedup! GmbH, grants from Pohl-Boskamp, grants from Inmunotek S.L., personal fees from John Wiley and Sons, AS, personal fees from Paul-Martini-Stiftung (PMS), outside the submitted work. Stefan A. Rudhart reports personal fees from UV-SMART outside the submitted work. Boris A. Stuck has received financial support for research, consultant and speaker fees from the following companies: G. Pohl-Boskamp GmbH & Co.KG, GlaxoSmithKlinie GmbH & Co KG and R. Cegla GmbH & Co.KG. He has also received financial support (sponsorship) for events of the Department of Otorhinolaryngology, Head and Neck Surgery, Marburg from: ALK Abello, Merck, Pohl Boskamp, Bristol Myers Squibb, Sanofi Genzyme. The other authors do not report any conflicts of interest. 

**List of abbreviations. Abbreviation:** List of abbreviations.

AIT	Allergen immunotherapy
AR	Allergic rhinitis
ARC	Allergic rhinoconjunctivitis
DBPC	Double-blind placebo-controlled
DNSSS	Daily nasal symptom severity score
IL-4	Interleukin 4
IL-13	Interleukin 13
IAR	Intermittent allergic rhinitis
MAB	Monoclonal antibodies
OCSS	Ocular symptom severity score
OJTF	Omalizumab joint task force
PAR	Perennial allergic rhinitis
RCT	Randomized controlled trials
RKI	Robert Koch Institute
RQLQ	Rhinoconjunctivitis quality of life questionnaire
SAR	Seasonal allergic rhinitis
SCIT	Subcutaneous AIT
TLR7	Toll-like receptor-7

**Table 1.** Clinical development programs of biologics in allergic rhinitis (most advanced study phase reported).

Substance name	Mechanism of action	Indication	Study phase	References	Manufacturer
Talizumab	IgE – antagonist	*	I	1997 [18]	Tanox
MEDI4212	IgE – antagonist	*	I	2016 [13]	MedImmune LLC
Ligelizumab	IgE – antagonist	A	I	2014 [14]	Novartis
PF-06444753 and PF-06444752	AHD and AHD+TLR-9 agonist, vaccine inducing anti-IgE antibodies	*	I	2015**	Pfizer
XmAb7195	IgE – antagonist	*	I	2017**	Xencor, Inc. ICON Early Phase Services, LLC
MT-2990	MAB***	*	I	2018**	Mitsubishi Tanabe Pharma Corporation
VAK-694	MAB against interleukin 4	*	IIa	2011 [15]	Novartis
Dectrekumab	MAB against interleukin-13	*	II	2008**	Novartis
GSK2245035	TLR-7 agonist	*	II	2015 [17]	GlaxoSmithKine
Dupilumab	MAB against IL-4 and IL-13 receptor	A, AD, CRS	II	2019 [16]	Regeneron Pharmaceuticals
Omalizumab	MAB against IgE	A, CSU	III	2018**	Novartis

A = asthma; AD = atopic dermatitis; AHD = aluminium hydroxide; CRS = chronic rhinosinusitis; CSU = chronic spontaneous urticaria. *Indication pending; **trial results (full publication) unpublished; ***MAB with undefined target.
